# Chemical Profiles of *Heterodera glycines* Suppressive Soils in Double Cropping Soybean Production

**DOI:** 10.2478/jofnem-2023-0030

**Published:** 2023-08-29

**Authors:** Leonardo F. Rocha, Mary E. Kinsel, Jason P. Bond, Ahmad. M. Fakhoury

**Affiliations:** School of Agricultural Sciences, Southern Illinois University, 1205 Lincoln Dr., Carbondale, IL, 62901, USA; School of Chemical and Biomolecular Sciences, Southern Illinois University, 1245 Lincoln Dr., Carbondale, IL, 62901, USA

**Keywords:** *biochemistry*, gas chromatography-mass spectrometry (GCMS), *Glycine max*, *Heterodera glycines*, host-parasitic relationship, management, metabolomics, suppressive soil

## Abstract

We previously reported soybean fields double-cropped with winter wheat having reduced soybean cyst nematode (SCN) (*Heterodera glycines*) counts compared to fallow. A follow-up metagenomics study identified several fungal and bacterial taxa enriched in wheat fields, and some were reported to parasitize SCN. Knowing that phytocompounds with potential nematicidal activity are released via wheat roots and stubble, we implemented a dichloromethane-based extraction method and a gas chromatography-mass spectrometry (GCMS) system to investigate soil chemical profiles of samples collected from these fields and review the potential nematicidal activity of compounds with higher concentration in double cropping fields. 51 compounds were detected during the GCMS analysis, eight with unknown identification. Several compounds, including multiple fatty acids, had larger relative peak areas when double-cropped, compared to fallow samples. This study, along with our previously published one, provided a better understanding of the mechanisms that govern the effect of wheat on SCN populations. Rather than driven by a single mechanism, the suppression of SCN in soybean fields double-cropped with winter wheat was potentially linked to enriched microbial communities, increased populations of beneficial organisms, and higher concentrations of chemicals with potential nematicidal activity. To our knowledge, this is the first study using GCMS to characterize soil chemical profiles in soybean fields double-cropped with winter wheat regarding the suppression of SCN populations.

Many plant-parasitic nematode species threaten soybean production worldwide, but the soybean cyst nematode (SCN) (*Heterodera glycines* Ichinohe) is by far the major problematic species in the Midwestern US (Bradley et al., 2021; Rocha et al., 2021a). A comprehensive selection of SCN management practices is recommended to minimize yield losses, including the use of resistant varieties, crop rotation with non-hosts, weed management, seed-applied nematicides, and biologically-based control methods (Niblack, 2005; Niblack et al., 2008; Rocha et al., 2021a; Nissan et al., 2022).

Double-cropping is defined as producing more than one crop on the same parcel of land in a single growing season (Rocha et al., 2021). In Southern Illinois, double-cropped soybean is commonly planted in fields following the harvest of winter wheat in mid-to-late June (Nafziger, 2009; Rocha et al., 2021). Double-cropping (DC) soybean with winter wheat has the potential to suppress SCN populations (Rocha et al., 2021b), as we previously reported soybean fields double-cropped with wheat having reduced SCN counts compared to fallow strips at the R1 stage (beginning of flowering) (−31.8%) and after soybean harvest (−32.7%) (Rocha et al., 2021b). In a follow-up study, a metagenomic analysis revealed marked contrasts in fungal communities across wheat and fallow fields throughout the soybean growing season, with several fungal and bacterial taxa being enriched in wheat fields (Rocha et al., 2022). Several enriched fungal and bacterial taxa in wheat plots, including *Mortierella, Exophiala, Conocybe, Rhizobacter* spp., and others, were previously reported to parasitize SCN and other plant-parasitic nematodes, suggesting a potential role of beneficial microbes in the suppression of SCN in soybean fields double-cropped with wheat. Further, the literature supports that a composite of elements, including environmental factors, the influence of wheat stubble, wheat root exudates, and mechanical interference with host recognition by SCN, contribute to reduced SCN population densities where wheat preceded soybean (Baird and Bernard, 1984; Koenning, 1991; Hershman and Bachi, 1995; Long and Todd, 2001; Warnke et al., 2006; Wight et al., 2011; Bernard, 2018).

Nematode-suppressive soils are characterized by low disease pressure even though both a plant-parasitic nematode and a crop host are present, and are linked to a series of soil biotic and abiotic factors (Cook and Baker, 1983; Mazzola, 2002). The suppressive effect is often connected to biofumigation, beneficial microorganisms competing for space and resources, mycoparasitism, direct antibiosis, the activation of plant defense mechanisms, and soil physiology modifications that create unsuitable conditions for nematode reproduction (Oka, 2010; Jung et al., 2012; Pimentel et al., 2020). Biofumigation is defined as the release of volatile compounds with pesticidal properties against weeds, nematodes, and plant pathogens from the breakdown of plant or animal materials (Larkin et al., 2012; Youseff, 2015). Phytochemicals in crop residue (stubble) and root tissues may contribute to biofumigation. Still, root exudates may be more critical through the actual discharge of such chemicals by living roots into the growth environment (Wu et al., 2001).

Root exudates are essential for shaping root-associated microbiomes, as they serve as a communication bridge between roots and surrounding organisms (Wang et al., 2021). In brassicas, which are commonly used as cover crops, volatile and toxic isothiocyanates formed via hydrolysis of secondary metabolites (glucosinolate compounds), organic acids, nitrogenous compounds (e.g., ammonia), or other plant secondary metabolites released during decomposition are reported to be involved in nematode suppression by interfering with their reproductive cycle (Termorshuizen et al., 2011; Hoorman, 2011; Mocali et al., 2015; Mashela et al., 2017; Dutta et al., 2019). Low-molecular-weight organic acids are involved in nematode suppression in rye fields, as these organic acids were detected in soil solutions. However, they are not solely responsible for nematode suppression, as they are rapidly degraded (McBride et al., 2000; Kaspar and Singer, 2011). In grasses, such as sorghum and sudangrass, cyanides produced *via* the enzymatic hydrolysis of precursor cyanogenic glycoside/dhurrin also have a nematicidal activity (Dutta et al., 2019). The effectiveness of these compounds in suppressing nematodes will depend on the physical and chemical properties of the soil, such as pH and texture (Termorshuizen et al., 2011).

Following wheat harvest, a layer of residue (wheat stubble) is left, decomposing over the soil throughout the soybean growing season. Wheat root exudates were shown to drive bacterial community structure and composition in the rhizosphere of watermelon, indicating that plant root exudates play a role in microbe recruiting and plant-to-plant signaling (Shi et al., 2021). Knowing that phytocompounds with potential nematicidal activity are released via the root systems of plants, the objectives of this work were to: *i*) summarize the organic chemical profile of fallow and double-cropping fields, *ii*) identify differences in the organic chemical profile between fallow and wheat plots, and *iii*) study seasonal variations in the release of phytochemicals via wheat stubble throughout the season.

## Material and Methods

### Field experimental design

Field trials were conducted from 2017 to 2018 to assess the effect of wheat on SCN population densities in DC soybean fields. Research plots were established in row crop fields located on commercial farms in Illinois, better simulating environmental conditions experienced by growers. In each location, the experimental design consisted of two treatments (WT: winter wheat; FL: fallow) with three replications. Each treatment was in strips (9.14 m wide × 182.9 m long), with three strips assigned for winter wheat and three strips that remained fallow. Strips were subdivided into three sub-plots (9.14 m wide × 61.0 m long for soil sampling). Thus, the study consisted of a total of 18 subplots with a total of 1.1 hectares per location (Figure 2). Wheat was planted in WT plots in Fall 2017, while fallow (FL) plots were maintained empty and weed-free over winter (Rocha et al., 2021a) until soybeans were planted in all subplots following the wheat harvest. Details regarding field site selection, experimental design, cropping practices, and sampling methodologies are available in Rocha et al. (2021b).

From each location, 18 soil samples were collected (two treatments × three replicates × three sub-samples per replicate) for each of the four sampling intervals. Samplings were conducted at the wheat establishment (1: November 2017), post-wheat/pre-soybean (2: June 2018), mid-soybean or R1 growth stage of soybean, which denotes the beginning of flowering (3: August 2018), and post-soybean harvest (4: October 2018).

### Sample selection for the GCMS analysis

Field locations with contrasting differences in SCN egg densities between wheat and fallow plots were previously selected for a metagenomics study (Rocha et al., 2022). All samples were ranked by the ratio of final (Pf - at soybean harvest - Oct. 2018) to initial SCN population density (Pi - at soybean planting - Jun. 2018). Locations with more distinguished differences in population ratios among WT and FL plots over this period were selected. In these locations, SCN population densities sharply increased in FL plots but were stable in WT plots. These included fields 3 (Nashville), 6 (Towers), and 8 (Oakdale), all located in Washington County, Illinois. From the original 18 samples collected from each field location (nine WT; nine FL), a total of 10 were selected: 5 with the most significant reductions in SCN counts (5 WT) and 5 with the sharpest increases in counts (5 FL). Location descriptions, including counts throughout field trials, are listed in Rocha et al. (2021b). For each plot selected, soil samples from three time points were included: post-wheat/pre-soybean, mid-soybean (R1 or beginning of flowering), and post-soybean.

The original metagenomics study included 90 soil samples: three fields × two treatments (wheat and fallow) × three time points (pre-, mid-, and post-soybean) × five replications. For the current GCMS analysis, all five replicates representing a location/treatment/timepoint were merged to create one 250 mg composite soil sample (50 mg from each replicate) to a total of 18 composite samples (three fields × two treatments × three time points). Samples were merged following the methodology of Ens et al. (2009). Soil samples were air-dried at room temperature for 24 hours before extraction.

### Sample preparation and GCMS analysis of organic extracts

The methods implemented during sample preparations and GCMS analysis were adapted from Ens et al. (2009). Composite soil samples (250 mg) were mixed with 500 ml of dichloromethane (Sigma-Aldrich – St. Louis, MO, USA) in 1000 ml autoclaved glass Erlenmeyer flasks. The soil and dichloromethane solution was mixed at room temperature in an orbital shaker for 24 h at 150 RPM. The supernatant (400–500 ml) was filtered through an autoclaved Watman 3 filter paper and concentrated into 2 ml using a rotary evaporator (BUCHI 011 Rotavapor™ Evaporator – New Castle, DE, USA). Samples were transferred to 2 ml clear glass screw thread vials (Thermo Fisher Scientific – Waltham, MA, USA), wrapped in aluminum foil, and stored at 4°C until GCMS analysis was performed.

A 1-ml aliquot of each soil extract was reacted with 100-ul of *N*, *O*-bis(trimethylsilyl)trifluoroacetamide (BSTFA, Supelco – Bellefonte, PA, USA) at room temperature for one hour. This step derivatized the fatty acid and sterol hydroxyl groups forming trimethyl silane esters, which improved compound volatility and chromatography. Derivatized soil extracts were then injected (1-ul) into a TraceGCultra PolarisQ gas chromatograph-mass spectrometer (Thermo Scientific - Waltham, MA, USA). The separation was performed using a DB5 non-polar capillary column (30 m × 0.25 mm × 0.25 um) (Agilent Technologies – Santa Clara, CA, USA). The GC inlet was held at 250°C with a split flow of 20:1 ml/min. The helium flow rate was 1.0 ml/min. The initial oven temperature was held at 80°C for 1 min, increased at 4°C/min to 100°C, then increased by 10°C/min to 280°C and held at 280°C for 10 min. The oven temperature was then increased at 10°C/min to 300°C and held for 44 min to ensure no carryover between soil extracts. The transfer line was maintained at 275°C. Collection of 70-eV electron impact mass spectra proceeded, and the total ion current was plotted as a function of time to yield each chromatogram. The retention times of a C7 – C40 saturated alkane standard (Millipore Sigma – Burlington, MA, USA) were used to calculate the retention index of compounds in soil extracts based on Kovats/Lee retention indices. Compound identifications were made by matching electron impact mass spectra and the retention indices to those published in the NIST20 database (2020).

### Data analysis

GCMS datasets were summarized and prepared for further analysis. Principal component analysis (PCA) based on singular value decomposition (SVD) was performed to compare the chemical profiles of soil samples across samplings (pre-soybean, mid-soybean, and post-soybean) and treatments (fallow or wheat) within a location. PCA was performed using ClustVis (Metsalu and Vilo, 2015).

The chemical profiles of soil samples collected from different locations (Nashville, Oakdale, and Towers) and samplings (pre-soybean, mid-soybean, and post-soybean) were compared across treatments using the following equation:

$$\mathrm{CR}=\left(\frac{\mathrm{WT}}{\mathrm{FL}}\right)\times 100$$

Where CR represents the compound ratio (CR) between wheat (WT) and fallow (FL) plots in a field at a specific sampling. Compound ratios over 100 indicate a higher concentration of WT samples, whereas CRs lower than 100 designate higher concentrations of FL samples. Table cells were colored based on CR results (CR>100 in gray and CR<100 in white).

A final analysis was conducted to estimate the correlation between the relative peak area (RPA) of a compound or group of compounds with SCN log-transformed counts (eggs per 100 cm^3^ of soil). Because soil samples were merged for the GCMS study, SCN counts were summed across combined samples. The Pearson correlation coefficient was used in JMP^®^ Pro 16.0.0 (SAS Institute Inc., Cary, NC, United States). A list with all correlation coefficients and their respective p-values is available in Table S3.

## Results

**Figure 1:**
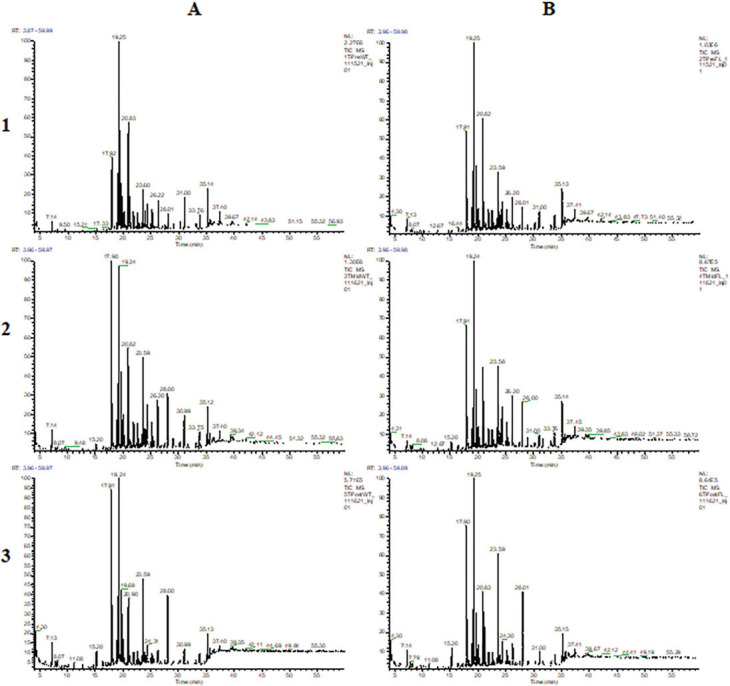
GCMS chromatograms of extracts from Towers soil samples collected from treatments (A: wheat; B: fallow) and time points (1: pre-soybean; 2: mid-soybean; 3: post-soybean).

**Figure 2:**
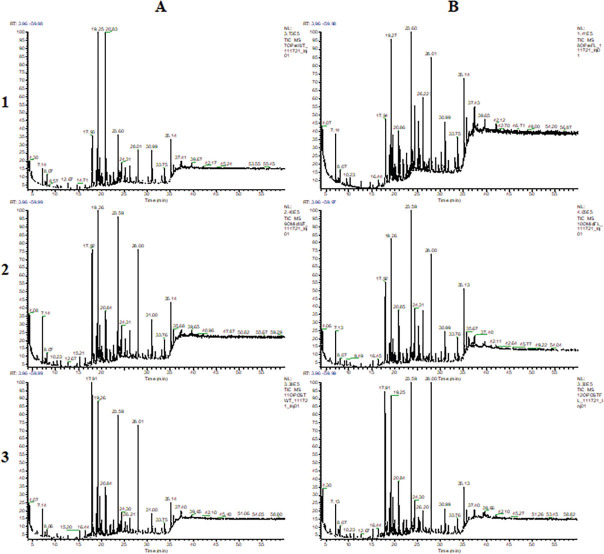
GCMS chromatograms of extracts from Oakdale soil samples collected from treatments (A: wheat; B: fallow) and time points (1: pre-soybean; 2: mid-soybean; 3: post-soybean).

**Figure 3:**
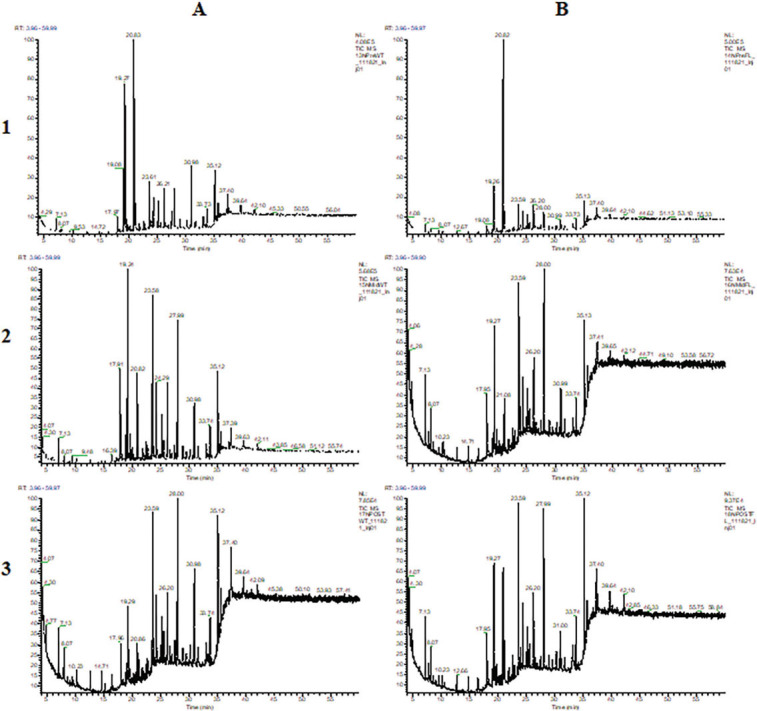
GCMS chromatograms of extracts from Nashville soil samples collected from treatments (A: wheat; B: fallow) and time points (1: pre-soybean; 2: mid-soybean; 3: post-soybean).

Detailed chromatograms are available for soil samples collected from Towers, Oakdale, and Nashville, in Figures 1, 2, and 3, respectively. Chromatograms are separated within locations by treatments (wheat and fallow) and time points (pre-, mid-, and post-soybean).

**Figure 4:**
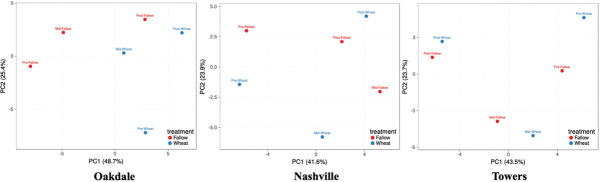
Principal component analysis (PCA) based on SVD comparing the chemical profiles of soil samples across time points (pre-soybean, mid-soybean, and post-soybean) and treatments (fallow or wheat) within a location (Nashville, Oakdale, and Towers). Analysis was performed using ClustVis (Metsalu and Vilo, 2015).

The soil chemical profile of samples collected in fields from time points (pre-soybean, mid-soybean, and post-soybean) and treatments (fallow or wheat) were analyzed using principal component analysis (PCA) within each location (Nashville, Oakdale, and Towers). For Oakdale, PC1 (X-axis) covered 48.7% of the variation, while PC2 (Y-axis) represented 25.4%. PC1 and PC2 in Nashville represented 41.6% and 23.9% of the total variation, respectively. In Towers, PC1 represented 43.5% of the variation, while PC2 was 23.7%. Samples’ chemical profiles tended to cluster by their respective treatments, but within treatments, samples were separated by sampling (Figure 4).

A total of 51 compounds were detected during the GCMS analysis, eight with unknown identification. Table S1 lists all compounds, retention times, retention indexes, database retention indexes, library matches, and their respective relative areas of compounds relative to the total hydrophobic extract area (Table S1). The number of compounds identified and their relative percent contribution of compounds (RA%) in different chemical functional groups are included in Table S2. Several compounds had greater compound ratios in WT samples compared to FL. Some compounds had a higher concentration in WT across all locations, while others had higher concentrations in specific samplings. The chemical profiles of soil samples collected from Nashville, Oakdale, and Towers at pre-, mid-, and post-soybean are displayed in Table 1.

**Table 1.** Comparison of chemical profile in soil samples collected from distinct locations (Nashville, Oakdale, and Towers) and samplings (pre-soybean, mid-soybean, and post-soybean). Numbers represent the ratio of a compound in wheat plots compared to fallow ((WT/FL)*100). Gray/white cells highlight higher compound concentrations in wheat or fallow plots under their respective fields and samplings.

**No.**	**Compound**	**Towers**			**Oakdale**			**Nashville**		
		
		**Pre-soy**	**Mid-soy**	**Post-soy**	**Pre-soy**	**Mid-soy**	**Post-soy**	**Pre-soy**	**Mid-soy**	**Post-soy**
1	Propylene glycol, 2TMS	67.47	112.52	112.58	144.03	126.04	71.9	0	63.77	84.46
2	Benzene, 1,3-bis(1,1-dimethylethyl)	64.31	112.28	123.28	0	80.49	70.98	43.56	75.45	96.67
3	Unknown	76.12	61.94	80.77	106.77	185.18	82.89	180.67	62.42	203.3
4	Tetradecanoic acid, TMS	110.09	69.67	186.51	-	181.16	122.92	-	-	-
5	Pentadecanoic acid, TMS	67.94	104.53	102.96	112.35	161.99	116.54	115.93	91.97	68.56
6	9-Hexadecenoic acid, TMS	144.23	111.39	126.21	247.56	174.87	139.98	325.52	282.77	107.57
7	Hexadecanoic acid, TMS	97.64	93.02	91.35	143.37	122.12	110.23	160.63	123.02	73.03
8	Cyclic octaatomic sulfur	-	-	-	-	-	-	-	-	-
9	Hetaptdecanoic acid, TMS	75.17	107.28	88.7	107.98	136.74	126.6	53.35	104.59	93.8
10	10-Heptadecenoic acid, (Z)-, TMS	74.04	133.24	102.98	124.76	130.28	118.56	136.32	113.68	75.51
11	9,12-Octadecadienoic acid, (Z,Z)-, TMS	148.82	106.39	110.56	570.09	89.22	78.11	59.52	-	10.26
12	9-Octadecenoic acid, (E)-, TMS	100.15	94.28	84.79	443.21	89.95	106.93	49.68	172.53	34.21
13	11-Octadecenoic acid, (E)-, TMS	79.83	109.79	103.17	223.7	134.89	138.75	58.25	29.32	12.39
14	Octadecanoic acid, TMS	98.92	78.44	77.55	147.03	110.22	106.14	86.05	86.39	74.22
15	10-Nonadecenoic acid, (Z)-, TMS	83.68	88.28	84.8	104.42	113.8	87.64	128.04	60.36	88.56
16	1-O-Pentadecylglycerol, 2TMS	72.98	108.39	90.66	129.44	134.23	92.56	141.3	33.56	48.12
17	1-Eicosanol, TMS	73.96	111.91	139.71	62.9	92.26	84.43	94.05	64.44	93.15
18	Unknown	95.75	124.82	110.63	87.27	95.56	112.57	113.83	72.35	103.82
19	Unknown	94.11	165.66	116.35	111.91	97.3	115.93	116	63.54	102.59
20	Icosanoic acid, TMS	98.79	128.39	108.26	47.48	76.04	136.91	76.73	94.16	90.82
21	2-Palmitoylglycerol, 2TMS	89.27	157.61	124.42	102.03	202.86	109.46	110.74	59.52	109.05
22	2-Palmitoleoylglycerol, 2TMS	105.35	149.11	104.52	72.17	120.79	142.35	109.14	67.75	77.69
23	Heneicosanoic acid, TMS	136.71	49.95	34.63	68.85	110.48	81.48	39.34	102.57	90.71
24	Docosanol, TMS	84.4	93.93	80.25	62.51	101.95	101.32	115.92	111.6	113.95
25	2-Mono-cis-heptdec-10-enoylglycerol, 2TMS	110.37	161.62	132.2	69.17	176.9	98.22	223.75	106.78	93.99
26	Behenic acid, TMS	92.31	89.1	79.2	54.7	55.35	78.55	105.28	119.97	117.57
27	Tricosanoic acid, TMS	95.15	89.93	73.56	49.2	53.26	77.58	95.38	69.74	106.89
28	Tetraconsanol acid, TMS	98.25	90.4	92.22	51.41	75.01	69.45	118.23	97.42	134.49
29	Lignoceric acid, TMS	98.78	84.4	59.98	37.79	52.1	80.02	93.93	95.9	101.25
30	Pentacosanoic acid, TMS	98.11	145.67	113.9	34.02	63.62	87.25	110.21	83.82	87.33
31	1-Hexacosanol, TMS	99.33	171.67	131.76	84.81	88.93	85.76	281.24	113.22	189.49
32	Tetracosanol, TMS	74.91	104.61	95.97	30.02	104.9	52.49	35.86	94.59	211.39
33	22-Hydroxydocosanoic acid, 2TMS	70.18	101.46	88.67	46.8	99.01	81.98	121.02	93.49	113.94
34	Hexacosanoic acid, TMS	106.56	79.67	74.82	45.11	59.11	69.42	138.89	98.13	149.95
35	Unknown	50.61	103.99	107.68	44.12	76.54	68.81	80.76	111.63	162.1
36	Unknown	117.77	159.57	173.24	75.8	99.34	100.51	76.72	58.22	93.97
37	Cholesterol, TMS	95.23	155.2	243.49	88.52	130.04	105.37	58.28	75.19	99.37
38	1-Octocosanol, TMS	179.08	223.9	133.62	104.99	113.58	95.96	326.9	139.31	235.07
39	24-Hydroxytetracosanoic acid, 2TMS	89.36	42.77	82.24	61.27	67.04	88.76	136	89.6	127.77
40	Unknown	78.66	65.32	96.83	58.68	78.03	100.82	80.88	25.97	90.2
41	Campesterol, TMS	108.82	80.77	116.27	102.73	98.05	76.07	106.11	98.38	111.96
42	Octacosanoic acid, TMS	112.96	131.88	92.38	45.85	73.59	73.42	161.76	130.73	200.57
43	Unknown	107.46	111.39	114.51	50.7	92.01	77.16	64.42	68.6	152.82
44	Stigmasterol, TMS	105.87	104.4	112.88	71.35	77.19	75.24	59.46	69.94	108.47
45	5alpha-Stigmast-7-en-3beta-ol, TMS	98.5	90.94	79.79	50.24	73.88	72.51	104.95	103.38	106.73
46	Unknown	112	317.17	98.08	94.92	96.4	316.59	78.24	74.68	178.13
47	Stigmast-5-ene, 3beta-TMS, (24S)-	108.91	78.49	96.98	78.74	63.32	74.47	119.09	97.77	86.97
48	Stigmastanol, TMS	94.13	99.07	160.9	68.09	68.95	70.67	107.33	65.1	180.53
49	1-Triacontanol, TMS	88	134.47	118.4	37.59	45.67	59.61	137.74	85.9	122.03
50	gamma-Sitostenone	74.83	136.96	116.28	41.02	143.39	102.62	89.31	98.91	64.9
51	Tricontanoate, TMS	122.73	96.25	66.52	39.05	47.58	62.35	100.78	87.35	159.01

In Towers 17, 30, and 27, compounds were detected with higher compound ratios (CR) in WT fields compared to FL at pre-, mid-, and post-soybean samplings, respectively (Table 1). The relative peak area of 9-hexadecenoic acid, 9,12-Octadecadienoic acid, 2-Palmitoleoylglycerol, 2-Mono-cis-heptdec-10-enoylglycerol, 1-Octacosanol, stigmasterol, unknown-36, and unknown-43 were consistently higher in WT samples, regardless of sampling. Compounds with the most elevated CR at pre-soybean included 1-octacosanol (179.08), 9,12-octadecadienoic acid (148.82), 9-hexadecenoic acid (144.23), heneicosanoic acid (136.71), and tricontanoate (122.73), while at mid-soybean, top compounds included unknown-46 (317.17), 1-octocosanol (223.90), 1-hexacosanol (171.67), unknown-19 (165.66), 2-palmitoyglycerol (157.61), 2-palmitoleoyglycerol (149.11) and pentacosanoic acid (145.67). In the later sampling (post-soybean), cholesterol (243.49), tetradecanoic acid (186.51), unknown-36 (173.24), stigmastanol (160.90), and 1-eicosanol (139.71) comprised the compounds with the highest CR in WT fields.

In Oakdale, 17, 21, and 20, compounds displayed higher CR in WT fields at pre-, mid-, and post-soybean samplings, respectively (Table 1). Notably, compound ratios of 9,12-octadecadienoic, 9-octadecenoic, 9-hexadecenoic, 11-octadecenoic, and octadecanoic acid were 570.09, 443.21, 247.56, 223.70, and 147.03 times at pre-soybean, respectively. Compound ratios of 2-Palmitoylglycerol, unknown-3, tetradecanoic acid, 2-Mono-cis-heptdec-10-enoylglycerol, and 9-Hexadecenoic acid were 202.86, 185.18, 181.16, 176.9, and 174.87 at mid-soybean, respectively. In the post-soybean sampling, compound ratios of 316.59, 142.35, 139.98, 138.75, and 136.91 were obtained for unknown-46, 2-Palmitoleoylglycerol, 9-Hexadecenoic acid, 11-Octadecenoic acid, and icosanoic acid, respectively. Furthermore, the overall CR of *n*-fatty acids decreased throughout the growing season but was consistently higher in WT fields (Table 2).

**Table 2.** Functional group profiles in soil samples collected from distinct locations (Nashville, Oakdale, and Towers) and samplings (pre-soybean, mid-soybean, and post-soybean). Numbers represent the ratio of the total relative area of a chemical in wheat plots compared to fallow ((WT/FL)*100). Gray/white cells highlight higher concentrations of a chemical group in wheat or fallow plots under their respective fields and samplings.

**Compound**	**Towers**			**Oakdale**			**Nashville**		

	**Pre-soy**	**Mid-soy**	**Post-soy**	**Pre-soy**	**Mid-soy**	**Post-soy**	**Pre-soy**	**Mid-soy**	**Post-soy**
*n*-fatty acids	93.23	96.39	93.61	124.33	110.72	106.24	89.82	104.37	78.57
saturated fatty alcohol	120.82	122.21	91.15	63.42	85.42	88.94	168.19	111.9	155.62
Sterols	106.29	88.63	110.96	77.96	72.72	75.63	108.38	93.67	100.14
Unknown	97.49	129.97	108.43	74.11	97.97	130.11	82.55	70.61	132.07
*n*-aldehydes	89.01	132.93	110	43.85	98.61	63.16	90.81	100	204.74
propane-1,2-diols	67.68	112.64	112.63	144.16	125.86	71.64	0	64.04	84.51
Steroid ketones	74.58	136.71	116.67	41.05	143.64	101.59	89.61	98.67	64.85
fatty acid glycerol	102.56	154.02	116.28	80.46	157.78	116.67	133.33	75	92.16
alkylbenzene	63.16	111.11	122.22	0	80.28	70.59	43.66	75	96.7

A total of 28, 14, and 26 compounds were identified with greater CR at pre-, mid, and post-soybean samplings in Nashville, respectively. Compounds with higher CR in WT compared to FL in Nashville at pre-soybean included 1-Octocosanol (326.90), 9-Hexadecenoic acid (325.52), 1-Hexacosanol (281.24), 2-Mono-cis-heptdec-10-enoylglycerol (223.75) and unknown-3 (180.67). At mid-soybean, compounds with the highest CRs included 9-Hexadecenoic acid (282.77), 9-Octadecenoic acid (172.53), 1-Octocosanol (139.31), octacosanoic acid (130.73), exadecenoic acid (123.02). Lastly, 1-Octocosanol (235.07), tetracosanol (211.39), unknown-3 (203.30), octacosanoic acid (200.57), 1-Hexacosanol (189.49), and stigmastanol (180.53) had the highest compound rations at post-soybean. Compound ratios of palmitelaidic acid, docosanol, behenic acid, 1-Hexacosanol, 1-Octocosanol, octacosanoic acid, and 5alpha-Stigmast-7-en-3beta-ol were greater in WT across all samplings in Nashville.

Notably, 9-Hexadecenoic acid (palmitelaidic acid) was present in higher concentrations in WT samples in all locations and samplings (CR>100). Out of the nine samplings (three locations × three timepoints), CRs of 1-Octocosanol were >100 eight times, several times for 10-Heptadecanoic and 2-palmitoyglycerol and six times for pentadecanoic acid, unknown-19, 2-Palmitoleoylglycerol, and 2-Mono-cis-heptdec-10-enoylglycerol.

In Nashville, higher concentrations of saturated fatty alcohols were observed in WT fields, while fatty acid glycerols were more abundant in Towers WT fields (Table 2). A series of compounds were negatively correlated with SCN soil counts, including Stigmastanol (R^2^: −0.6352; P=0.0046), 1-Hexacosanol (R^2^: −0.4777; P=0.045), Unknown-43 (R^2^: −0.4511; P=0.0603), Benzene, 1,3-bis(1,1-dimethylethyl) (R^2^: −0.44; P=0.0677), 1-Octocosanol (R^2^: −0.427; P=0.0772), Icosanoic acid (R^2^: −0.4208; P=0.082), Campesterol (R^2^: −0.4202; P=0.0825), Pentacosanoic acid (R^2^: −0.4117; P=0.0896), 5alpha-Stigmast-7-en-3beta-ol (R^2^: −0.4056; P=0.0949), and Tetracosanol (R^2^: −0.4027; P=0.0975 (Table S3). Chemical groups were also negatively correlated with SCN counts, comprising saturated fatty alcohols (R^2^: −0.4926; P=0.0378), n-alde hydes (R^2^: −0.472; P=0.0479), alkylbenzenes (R^2^: −0.4394; P=0.0603) unknown compounds (R^2^: −0.472; P=0.077), and sterols (R^2^: −0.3974; P=0.1024) (Table S3).

## Discussion

Field trials were conducted from 2017 to 2018 to investigate the effect of wheat on SCN populations in double-cropping soybeans. Wheat was planted in strips alternating with strips that were kept weed-free and fallow over winter and early spring. Soybeans were planted in all strips after the wheat harvest. Wheat strips had reduced SCN egg densities compared to fallow at the R1 stage (−31.8%) and after soybean harvest (−32.7%) (Rocha et al., 2021b). A later metagenomics study unraveled substantial differences in fungal communities across wheat and fallow fields throughout the soybean growing season. Several fungal and bacterial taxa were enriched in wheat fields (Rocha et al., 2022). The current study investigates soil chemical profiles from these fields and reviews the potential nematicidal activity of compounds with higher concentrations in double-cropping soybean fields.

An array of plant secondary metabolites is released via plant roots and decomposing crop residue, including phenols, terpenoids, alkaloids, coumarins, tannins, flavonoids, steroids, and quinines (Chitwood, 2002; Lam et al., 2012). The current analysis identified 51 compounds, including several fatty acids, some of which were found to have increased concentrations in soil samples double-cropped with winter wheat. Most of these compounds are reported in the literature as being significant constituents of wheat straw, while others are also produced by the soybean crop, including Hexadecanoic acid and its derivatives (Alghamdi et al., 2018). In wheat straw, the literature reports long-chain free fatty acids, free fatty alcohols, long-chain fatty alcohols, aliphatic series, β-diketones, and steroid compounds as the major lipids in wheat (del Rio et al., 2013). del Rio et al. (2013) reported n-hexadecanoic, octadec-9-enoic, n-octacosanoic, octadeca-9,12-dienoic and n-docosanoic acids as the five most abundant fatty acids in wheat straw. San-Emeterio et al. (2021), using a combination of analytical pyrolysis coupled with gas chromatography-mass spectrometry (Py-GC/MS), identified tetradecanoic acid, hexadecanoic acid, 11-octadecenoic acid, octadecanoic acid, docosanoic acid, tricosanoic acid, tetracosanoic acid, stigmastanol and gamma-sitostenone in composting maize biomass.

The nematicidal activity of fatty acids, abundant in our analysis, often depends on chain length and the number and position of double bonds (Faizi et al., 2011). 9-Octadecenoic acid (oleic acid), 9,12-Octadecenoic acid (linoleic acid), and 11-Octadecenoic acid (vaccenic acid) are abundant and naturally occurring fatty acids. Stadler et al. (1994) reported multiple fatty acids and other compounds isolated from cultures of Basidiomycetes with nematicidal activity against *Caenorhabditis elegans*. The compounds were similarly identified in our soil samples and their respective reported LD_50_ (μg/mL) in the literature are: tetradecanoic acid (5); hexadecanoic acid (25), octadecanoic acid (50), icosanoic acid (100), 9Z)-hexadec-9-enoic acid (10), 9-Octadecenoic acid (25), 9,12-Octadecadienoic acid (5), 9-Octadecenoic acid, and 9,12-Octadecadienoic, octadecanoic, hexadecanoic, and tetradecanoic acids, which significantly controlled *Heterodera zeae* (corn cyst nematode) under in-vitro conditions (Faizi et al., 2011; Zhang et al., 2012). 9,12-Octadecadienoic acid was shown to be responsible for the nematicidal activity of the Oken fruit (*Holigarna caustica* Dennst.) against *C. elegans* (Panda et al., 2020), and it is also released from the mycelia of *Arthrobotrys* spp., which are known nematode control agents (Stadler et al., 1993). 10-endecanoic acid proved effective in controlling egg masses of the root-knot nematode (*Meloidogyne incognita*) (Tarjan and Cheo, 1956). Heptanoic, octanoic, nonanoic, decanoic, and undecanoic acids demonstrated nematicidal activity against *Panagrellus redivivus* and reduced or stopped egg hatching in *Heterodera tabacum* (Tarjan and Cheo, 1956). Hexanoic, octanoic, decanoic, dodecanoic, tetradecanoic, and hexadecanoic acids caused significantly higher mortality in *M. incognita* J2s than other fatty acids. Both octanoic and decanoic acids resulted in approximately 50% nematode mortality after a 24 h exposure (Zhang et al., 2012).

Palmitoylglycerol, which had increased relative peak areas across WT fields, has known antibacterial activity (Adnan et al., 2019). Tricontanoate is another compound identified at higher concentrations following the wheat harvest in two fields (Towers and Nashville). While dosocanol is reportedly the major policosanol in wheat, tricosanol, tetracosanol, hexacosanol, octacosanol, and triacontanol are also commonly found, even across different wheat varieties (Irmak and Dunford, 2005; Chen et al., 2009). Nogueira et al. (1996) reported that triacontanol and tricontanyl tetracosanoate extracted from leaves and roots of *Mucuna aterrima* suppressed *M. incognita* egg hatching. Triacontanol has also been shown to improve plant growth and physiological activities in diverse groups of plants, even when applied at a nanomolar concentration (Naeem et al., 2012).

Further research is needed to explore the role of these compounds, solo or as a mixture, on SCN suppression. Although these compounds could be tested individually for their nematicidal activity, research suggests that an array of compounds acting collectively instead of individual compounds are more likely to suppress nematodes in field conditions (Huang et al., 2003). The biological activity of a group of phytochemicals may produce a more substantial effect, equivalent to a much higher concentration of the most active compound. A limitation of our study was using only three field locations out of the original nine and the sample merging, which resulted in the lack of replicates. In addition, inherently, the extraction methodology and the GCMS approach target specific groups of compounds. Despite these limitations, this work provides a basic profile of chemicals in soil samples from soybean fields double-cropped with winter wheat. To our knowledge, this is the first study using GCMS to characterize soil chemical profiles in soybean fields double-cropped with winter wheat regarding suppression of SCN populations.

In the current study, GCMS analysis reveals distinct soil chemical profiles across wheat and fallow fields throughout the soybean growing season, highlighting compounds in WT samples and their potential nematicidal activity. This study, along with our previous publications (Rocha et al., 2021b; Rocha et al., 2022), provide a better understanding of the mechanisms that govern the effect of wheat on SCN populations. Rather than a single mechanism, the suppression of SCN in soybean fields double-cropped with winter wheat is potentially linked to enriched microbial communities, increased populations of beneficial organisms, and higher concentrations of phytochemicals with nematicidal activity.

## Supplementary Material

Supplementary Material Details

## Abbreviations

SCN: soybean cyst nematode
GCMS: gas chromatography-mass spectrometry
WT: wheat
FL: fallow
CR: compound ratio
RPA: relative peak area
PC: principal component
PCA: principal component analysis
